# Correction of center of rotation and projection angle in synchrotron X-ray computed tomography

**DOI:** 10.1038/s41598-018-28149-8

**Published:** 2018-06-29

**Authors:** Chang-Chieh Cheng, Yu-Tai Ching, Pai-Hung Ko, Yeukuang Hwu

**Affiliations:** 1 Department of Computer Science, College of Computer Science, National Chiao Tung University, 1001 University Road, Hsinchu, Taiwan; 2 Department of Engineering Science, National Cheng Kung University, No. 1, University Road, Tainan, Taiwan; 3 Institute of Physics, Academia Sinica, 128 Academia Road, Nankang, Taipei Taiwan

## Abstract

An error in tomographic reconstruction parameters can result considerable artifacts in the reconstructed image, particularly in micro-computed tomography and nano-computed tomography. This study involved designing an automatic method for efficiently correcting errors resulting from incorrectly determined rotational axes and projection angles. In this method, errors are corrected by minimizing the “total variation” of a reconstructed image, and minimization is accomplished by using the gradient descent method. Compared with two previous methods, the proposed method achieved the best reconstruction results.

## Introduction

Synchrotron X-ray computed tomography (SXCT) uses synchrotron radiation comprising high collimation and low diffraction X-ray beams as the light source for micro-computed tomography and nano-computed tomography^1^. The main advantage of SXCT is the high resolution and nondestructive visualization of the interior of objects. SXCT has been widely applied in biology studies and industrial applications such as visualization of tumor growth^2^ and nanofabrication^3^.

Compared with images acquired using a well-calibrated CT scanner in a hospital, those acquired through SXCT more likely contain artifacts such as ring artifacts caused by imperfections in the detector^4^. In SXCT, the object rotates around a vertical axis to acquire projections from different angles. This image acquisition process considerably increases the introduction of errors into the reconstruction steps, and produces severe artifacts if the reconstruction parameters are not accurate. For example, tuning-fork artifacts are caused by errors in a reconstruction parameter^5^. Because a rotating object is required, the mechanical instability of the holder is also a major problem when images are at the nanoscale^6^. In the worst case, no reconstruction is possible.

This paper proposes a method that suppresses the tuning-fork artifacts caused by biases of the rotational axis. As mentioned, the light source of SXCT is fixed; the object holder rotates around a vertical axis to acquire projections from different angles to construct a sinogram. The vertical line at the center of the sinogram should be the projection of the rotational axis; however, accomplishing this is highly difficult, particularly with high-resolution image acquisition. Although such inaccuracy can hardly be corrected through hardware improvement, but it can be resolved by using computer methods. Two methods has been reported to address this problem: entropy-based correction (ENP)^7^ and frequency-based correction (FC)^8^. The ENP method uses entropy^9^ to define a metric of reconstruction quality. The best possible reconstruction can be obtained by iteratively testing different biases of the rotational axis. The reconstruction that minimizes the metric is considered the best reconstruction. If the projections are parallel and considered over a half range (i.e., from 0° to 180°), the FC method estimates the bias in the frequency domain of the sinogram. The biases of the rotational axis also occurred in clinical tomography with full range scan (i.e., from 0° to 360°)^10,11^. There are many methods based on Helgason—Ludwig consistency condition (HLCC) can correct the bias in the full-range scan^12–14^. However, since the energy of synchrotron X-ray is larger than 1 GeV, the half-range scan is commonly used in SXCT to reduce radiation dose. Another reason of the half-range scan in SXCT is that some containers of biological samples cannot be rotated over 180°, even less than 180°, otherwise, the structure of container will affect the projection of sample to produce unwanted reconstruction results. Therefore, SXCT requires a correction method for the projection data acquired by half-range scan. That is main motivation of the proposed method.

An angular error between two consecutive projections also causes artifacts in the reconstructed image. We present a phantom to explain these types of artifacts. Figure 1a shows the original image (the ground truth). Figure 1b shows the tomographic image of a phantom reconstructed from 600 projections with 1024 parallel beams, with the interval of the projection angles being 0.303°. Figure 1c shows the tomographic image reconstructed from the same projections but with its projection angles incorrectly configured to 0.3°; notable artifacts can be observed in the reconstructed image.

**Figure 1:**
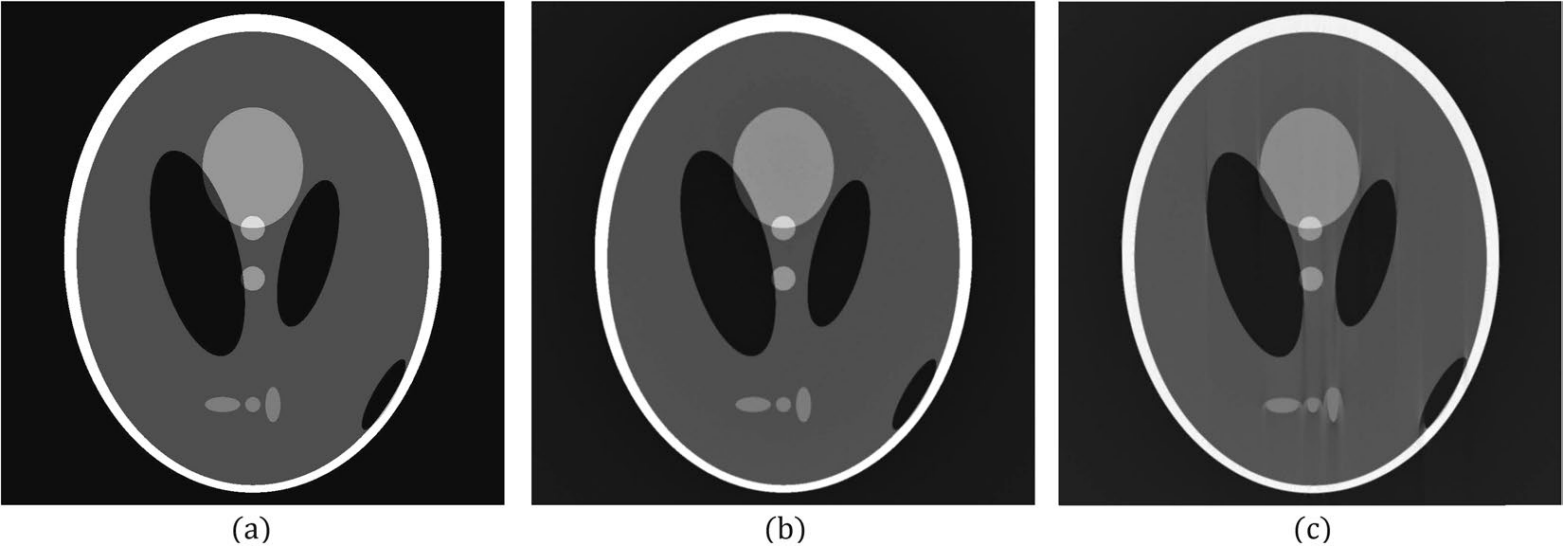
Tomographic images of a phantom reconstructed from 600 projections with 1024 parallel beams; the interval of the projection angles was 0.303°. (**a**) Original image. (**b**) Result obtained using correct reconstruction parameters. (**c**) Result obtained using an interval of 0.3° instead of 0.303°.

An iterative method similar to ENP can correct the projection angle errors. Suppose that the error is in the interval [*u*_*a*_, *u*_*b*_], *u*_*b*_ > *u*_*a*_. [*u*_*a*_, *u*_*b*_] can be digitized into *n* values, and each value is a candidate for the true projection angle. The desired reconstruction is achieved when the best reconstructed image is obtained with one of the digitized values. Depending on the value of *n* and the reconstruction algorithm, obtaining the best reconstruction could be a time-consuming task. This method is appropriate for correcting errors with a single parameter. If the number of parameters is *k* and the range of each parameter is divided into *n* values, *n*^*k*^ combinations of the reconstruction parameters can be derived. Nevertheless, the computing time renders this method impractical, even for recovering two parameters.

This paper presents a novel method that efficiently suppress artifacts caused by biases of the rotational axis and errors in the projection angle. Hereafter, we use *δ*_*B*_ and *θ*_*E*_ to denote these two types of errors, respectively. The proposed method uses the total variation^15,16^ as a metric for the quality of a reconstruction. The gradient descent method^17^ is then employed to minimize the total variation so that the parameters are corrected.

## Methods

### Statement

All experiments and methods were performed in accordance with relevant guidelines and regulations. All experimental protocols were approved by a named institutional/licencing committee. Specifically, all procedures involving the animals were approved by the Academia Sinica Institute Animal Care and Utilization Committee (AS IACUC). BALB/c mice were provided by National Laboratory Animal Center, Taiwan. All mice were housed in individual ventilated cages with wood chip bedding and kept at 24 ± 2 °C with a humidity of 40–70% and a 12-hour light/dark cycle.

### Reconstruction quality metric

The proposed method defines a metric to present the quality of the reconstructed image. The errors *δ*_*B*_ and *θ*_*E*_ are then computed by minimizing the quantity of the defined metric. Given an image with *n* × *m* pixels, let *I*(*x*, *y*) be the intensity of the pixel (*x*, *y*), *x* = 1, 2, …, *n* and *y* = 1, 2, …, *m*. The total variation, *TV*, is defined as in Eq. (1):

1 $$TV(I)=\sum _{y=1}^{m}\sum _{x=1}^{n}\,|I^{\prime} (x,y)|,$$

where

2 $$I^{\prime} (x,y)={[{I}_{x},{I}_{y}]}^{T}={[\frac{I(x+1,y)-I(x-1,y)}{2},\frac{I(x,y+\mathrm{1)}-I(x,y-1)}{2}]}^{T},$$

and

3 $$|I^{\prime} (x,y)|=\sqrt{{I}_{x}^{2}+{I}_{y}^{2}}.$$

According to the definition, *TV* is the sum over all pixels of squared differences from neighboring pixels. Note that Eq. (2) is an operator to enhance high-frequency signals (in particular, edge detection). Thus, *TV* is large if the image contains high-frequency signals such as noise and edges. Because a tomographic image with errors *δ*_*B*_ and *θ*_*E*_ contains artifacts of arcs and lines, *TV* is appropriate to measure the quality of tomographic reconstruction and serves as a guideline to correct any inaccurate reconstruction parameters. To ensure that *TV* is associated with the arc and line artifacts, a low-pass filter, such as a mean filter or a Gaussian filter, is applied to the images before *TV* is computed. In this study, a 7 × 7 Gaussian filter with a standard deviation of 0.84 was applied.

### Gradient descent

Consider a differentiable function *F*(**u**), where **u** = [*u*_1_, *u*_2_, $\cdots$, *u*_*k*_]^*T*^ in the range [**u**_*a*_, **u**_*b*_], *u*_*a*1_ < *u*_*b*1_, *u*_*a*2_ < *u*_*b*2_, $\cdots$, *u*_*ak*_ < *u*_*bk*_. If *F* is convex in the range [**u**_*a*_, **u**_*b*_], the minimum of *F* in the range [**u**_*a*_, **u**_*b*_] can be computed using the gradient descent method. The gradient descent recursion with *t* iterations can be written as

4 $${{\bf{u}}}^{i+1}={{\bf{u}}}^{i}-{\alpha }_{i}F^{\prime} ({{\bf{u}}}^{i}),$$

where 1 ≤ *i* < *t*, *α*_*i*_ is the step size of the *i*-th iteration, and

5 $$F^{\prime} ({\bf{u}})={[\frac{\partial F({\bf{u}})}{\partial {u}_{1}},\frac{\partial F({\bf{u}})}{\partial {u}_{2}},\cdots ,\frac{\partial F({\bf{u}})}{\partial {u}_{k}}]}^{T}.$$

As *i* approaches *t*, **u**^*i*+1^ in Eq. (4) moves toward the minimum. Let *Q* be an operator for computing the *TV* of an image reconstructed by a tomographic reconstruction algorithm *R* with a set of parameters **u**. Assume that **u** is within the range between **u**_*a*_ and **u**_*b*_; then, *Q* is defined as follows:

6 $$Q({\bf{u}})=TV(R({\bf{P}},{\bf{u}})),$$

where **P** is a set of X-ray projections. Then, we can substitute *Q*(**u**) into Eq. (4) to yield

7 $${{\bf{u}}}^{i+1}={{\bf{u}}}^{i}-{\alpha }_{i}Q^{\prime} ({{\bf{u}}}^{i}).$$

In Eq. (7), $Q^{\prime} ({{\bf{u}}}^{i})$ is estimated through numerical differentiation as in Eq. (8)

8 $$Q^{\prime} ({{\bf{u}}}^{i})=-\,\frac{1}{H}\sum _{h=1}^{H}\frac{Q({{\bf{u}}}^{i}+h{\rm{\Delta }}{{\bf{u}}}^{i})-Q({{\bf{u}}}^{i}-h{\rm{\Delta }}{{\bf{u}}}^{i})}{2h{\rm{\Delta }}{{\bf{u}}}^{i}},$$

where Δ**u**^*i*^ is the variation of **u**^*i*^, and 2*H* is the number of neighbors of **u**^*i*^. To estimate the tendency of convergence of *Q* in the range [**u**_*a*_, **u**_*b*_], Δ**u** should be large in the first iteration and should decrease as the number of current iterations increases. In this study, Δ**u**^1^ = (**u**_*b*_ − **u**_*a*_)/2*H*. In the (*i* + 1)-th iteration, for the *j*-th component of Δ**u**, ${\rm{\Delta }}{u}_{j}^{i+1}={\rm{\Delta }}{u}_{j}^{i}\mathrm{/2}$ if ${\rm{\Delta }}{u}_{j}^{i}$ is greater than Δ*v*_*j*_, which is the smallest variation of *u*_*j*_; otherwise, ${\rm{\Delta }}{u}_{j}^{i+1}={\rm{\Delta }}{u}_{j}^{i}$. The step size *α* is given by Eq. (9):

9 $${\alpha }_{i+1}=\{\begin{array}{cc}\frac{{\alpha }_{i}}{2} & {\rm{if}}\,\exists {\rm{\Delta }}{u}_{j}^{i} > {\rm{\Delta }}{v}_{j},\\ {\alpha }_{i} & {\rm{otherwise}}.\end{array}$$

In real application, the domain may not be convex, and Eq. (7) may not converge or may converge to a local minimal. To avoid divergence, the number of iterations is limited by a preset value *t*_*m*_. The search fails if *i* = *t*_*m*_ or any component of **u**^*i*^ is out of range. By contrast, the search terminates before *i* reaches *t*_*m*_ if *Q*′(**u**^*i*^) is a near-zero number and *α*_*i*_ = *α*_*i*−1_. In this case, Eq. (7) converges to the minimum or a local minimum. In the implementation, each of *H*, *α*_0_, and *t*_*m*_ should be assigned a reasonable value. Assigning *H* = 2 or 3, *α*_0_ = 1.0, and *t*_*m*_ = 20 is effective in most cases.

### Implementation

The gradient descent method requires an adequate range of [**u**_*a*_, **u**_*b*_] such that the solution falls within the range. The initial point, **u**^**0**^, for the recursion (Eq. 7) also affects the result of the gradient descent method^17^. Determining the most adequate range and **u**^**0**^ for each case is difficult. We propose a multi-range testing method to overcome this problem. The idea of this method is simple: We test several ranges with different **u**^**0**^, and the best result among all the tests is the solution to the problem. The implementation is listed as Algorithm 1.

**Algorithm 1:**
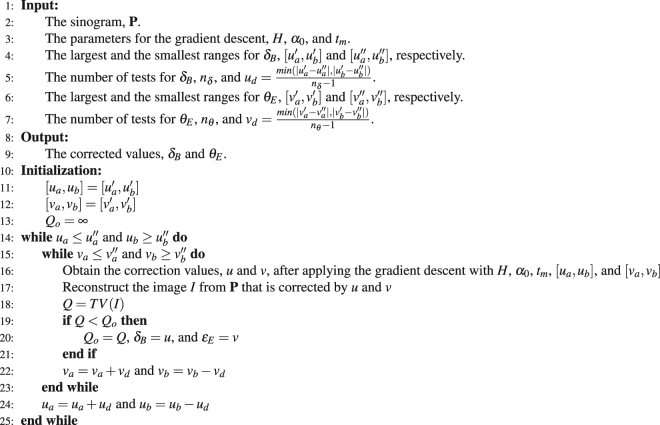
Multi-range testing.

In most cases, the largest and smallest ranges of *δ*_*B*_ are ±55 and ±10 pixels respectively. The largest and smallest ranges of *θ*_*E*_ are ±0.1% and ±0.01% of the projection angle. The ranges of *δ*_*B*_ and *θ*_*E*_ are digitized to 10 tests, where the differences between consecutive ranges of *δ*_*B*_ and *θ*_*E*_ are 2*u*_*d*_ and 2*v*_*d*_, respectively. Therefore, the total number of tests is 100. The test that achieves the least total variation, *Q*, yields the corrected *δ*_*B*_ and *θ*_*E*_.

### Experimental design

Two computer generated phantom images and a mouse kidney image were used in this experiment. Phantom images are often used as the ground truth to validate or compare tomographic reconstruction algorithms. In this study, the use of the phantom images was necessary because of the microscale or nanoscale resolution of SXCT; physically creating a phantom for ground truth is difficult or even impossible. Phantom 1 (Figs 1 and 2) was the Shepp-Logan phantom^18^, which is typically used to evaluate almost all reconstruction algorithms^19^. Because artifacts caused by *δ*_*B*_ and *θ*_*E*_ occur at places involving significant changes in intensity, we designed Phantom 2 (Fig. 3) to enhance the artifacts.

**Figure 2:**
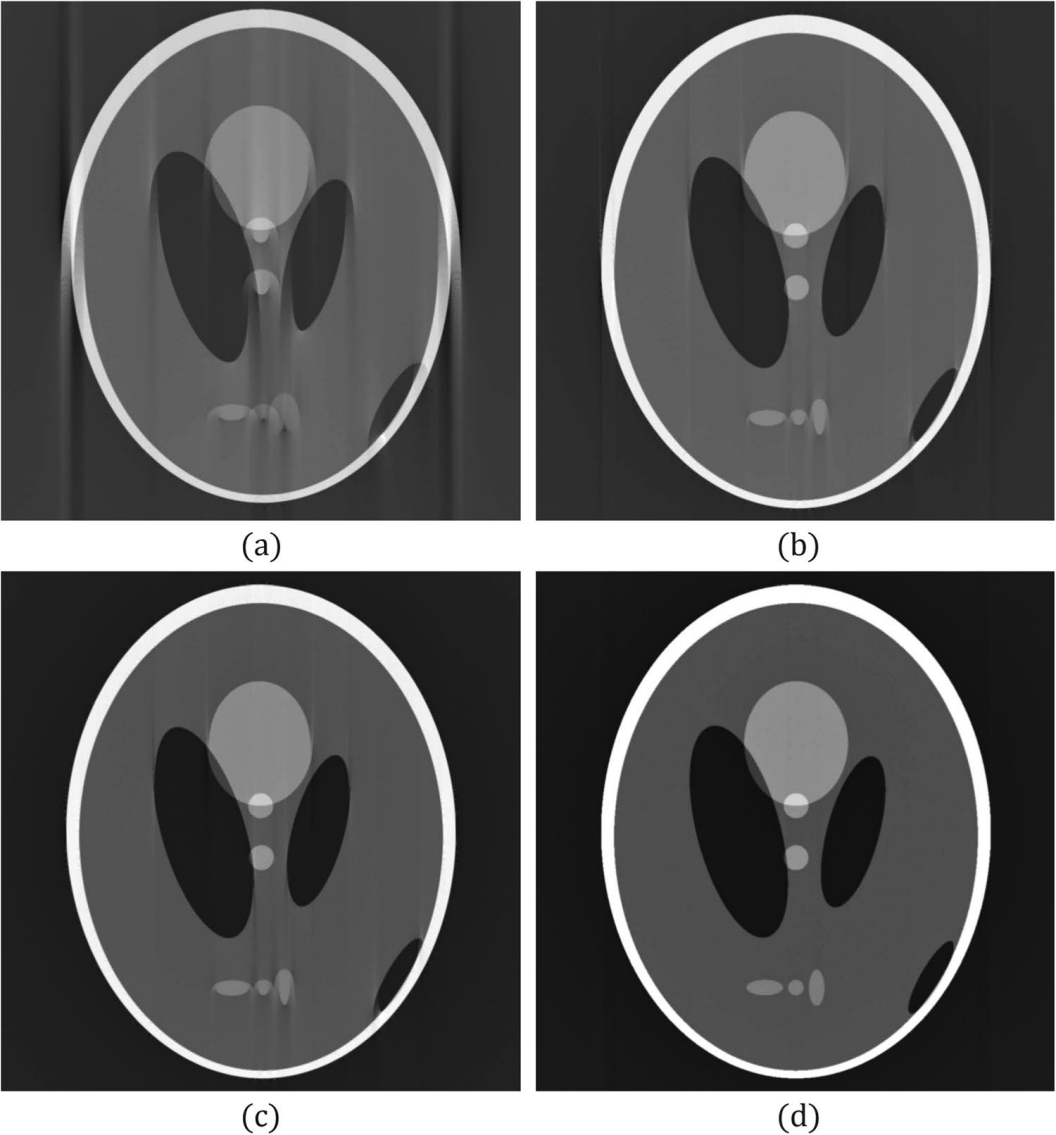
Tomographic images of Phantom 1 reconstructed from 600 projections with 1024 parallel beams. The interval of angle for generating the projections was 0.303° and the projected rotational axis was biased to the right by 10 pixels. (**a**) Reconstruction result without correction of the interval of projection angles or the rotational axis. (**b**) Reconstruction result when *δ*_*B*_ was corrected by the ENP method. (**c**) Reconstruction result when *δ*_*B*_ was corrected by the FC method. (**d**) Reconstruction result when *δ*_*B*_ and *θ*_*E*_ were corrected by the proposed method.

**Figure 3:**
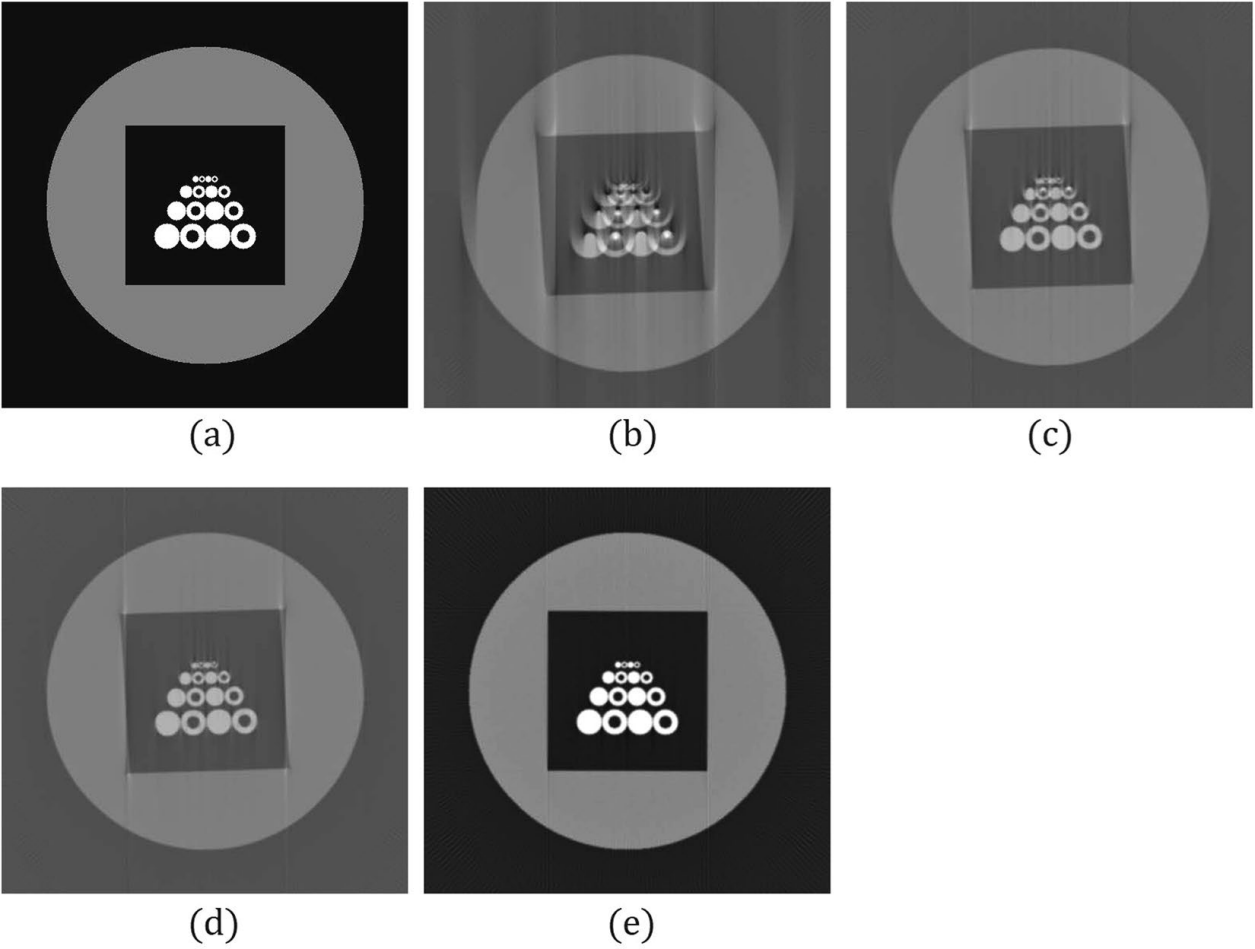
Tomographic images of Phantom 2 reconstructed from 180 projections with 512 parallel beams. The projection angle was 1.02° and the projected rotational axis was biased to the left by 10 pixels. (**a**) Target image. (**b**) Reconstruction result without correcting the errors. (**c**) Reconstruction result when *δ*_*B*_ was corrected by the ENP method. (**d**) Reconstruction result when *θ*_*E*_ was corrected by the FC method. (**e**) Reconstruction result when *δ*_*B*_ and *θ*_*E*_ were corrected by the proposed method.

The sinogram of Phantom 1 comprised 1024 × 600 projection data (1024 parallel beams × 600 projection angles), and the projection angles was 0.303° when the sinogram was generated. During the reconstruction, the angle was configured as 0.3° (i.e., *θ*_*E*_ = 1%). The sinogram was shifted toward the right by 10 pixels as *δ*_*B*_ (i.e., *δ*_*B*_ = +10). Figure 2a shows an image of 1024 × 1024 pixels reconstructed without correcting the biases.

Phantom 2 involved 16 white circles (with different radii) enclosed by a black square; the black square was inside a larger gray circle. The image size was 512 × 512 pixels. The sinogram of Phantom 2 comprised 512 × 180 projection data (512 parallel beams × 180 projection angles), and the projection angle was 1.02° when the sinogram was generated. During the reconstruction, the projection angle for reconstruction was configured as 1.0° (i.e., *θ*_*E*_ = 2%). The sinogram was shifted toward the left by 10 pixels as *δ*_*B*_ (i.e., *δ*_*B*_ = −10). Figure 3b shows an image reconstructed without correcting the biases.

The third test sample was a set of real data, a sinogram acquired from a mouse kidney using the facility at the National Synchrotron Radiation Research Center, Hsinchu, Taiwan. The vessel tissues were stained by barium sulfate (BaSO_4_). The sinogram comprised of 1600 × 601 X-ray projections (1600 parallel beams × 601 projections). The size of each pixel was 2.76 *μ*m^2^, and the projection angle of the instrument was recorded as 0.2995°.

The correction methods used were ENP, FC, and the proposed method. Because the ENP and FC could correct *δ*_*B*_ only for a given range that was set to ±20 pixels, we left *θ*_*E*_ un-corrected during the application of ENP and FC. However, the proposed method corrected both biases.

The reconstruction algorithm was the filtered back projection (FBP). An NVIDIA GTX 980 graphics processing unit (GPU) was used to accelerate the reconstruction^20^. A computer equipped with an Intel Xeon E3 CPU and 32 GB memory was used.

## Results

### Phantom 1

The results obtained by applying the ENP, FC, and proposed methods are summarized in Table 1. Because we had ground truth information, we could compute the mean squared error (MSE) of the ground truth with the reconstruction results. The parameters used for applying the proposed method were *H* = 2, *α*_0_ = 1.0, and *t*_*m*_ = 20. For multi-range testing, we set 10 ranges for *δ*_*B*_ from ±55 pixels to ±10 pixels (*u*_*d*_ = 5 pixels) and 10 ranges for *θ*_*E*_ from ±0.03° to ±0.003° (*v*_*d*_ = 0.003°). The multi-range testing required approximately 970 s, nearly 9.7 seconds for a range. A visual comparison of the reconstructed images revealed that the proposed method achieved the best result. The MSE between the ground truth and the reconstructed image provided by the proposed method was 0.002, which is also the best among the three methods.

**Table 1:** Results of applying the ENP, FC, and proposed methods to Phantom 1.

Method	*δ*_*B*_ (pixel)	*θ*_*E*_ (°)	MSE	Computing Time (sec.)	Reconstructed Image
ENP	11	—	0.021	3.9	Figure 2b
FC	10	—	0.009	4.6	Figure 2c
Proposed Method	10	0.303	0.002	970 (9.7 × 100)	Figure 2d

The *θ*_*E*_ values of the ENP and FC methods are denoted by dashes because these two methods could not correct the projection angle errors.

### Phantom 2

The reconstruction without any correction is shown in Fig. 3b. The results obtained by applying the ENP, FC, and proposed methods are summarized in Table 2. The parameters used in the proposed method were *H* = 3, *α*_0_ = 1.0, and *t*_*m*_ = 20. The proposed method had the least MSE.

**Table 2:** Results of applying ENP, FC, and the proposed method to Phantom 2.

Method	*δ*_*B*_ (pixel)	*θ*_*E*_ (°)	MSE	Computing Time (sec.)	Reconstructed Image
ENP	−8	—	0.076	1.4	Figure 3c
FC	−9.5	—	0.074	3.2	Figure 3d
Proposed Method	−10	1.0226	0.002	172 (1.7 × 100)	Figure 3e

### Mouse kidney

The final test sample was a slice of mouse kidney. Figure 4a shows a reconstructed image of 1600 × 1600 pixels without error corrections. Figure 4b presents the enlarged view of the region bounded by the white-bordered rectangle in Fig. 4a. The ENP and FC methods were applied to compute *δ*_*B*_. The ENP and FC methods shifted the rotational axis toward the right by 2 and 3.5 pixels, respectively, and they required 19.5 and 12.5 s, respectively. The reconstruction results obtained using the ENP and FC methods are shown in Fig. 5a and b, respectively. With the same view boundary as in Figs 4a and 5d,e show the enlarged views of Fig. 5a,b, respectively. Improvements were observed after correction.

**Figure 4:**
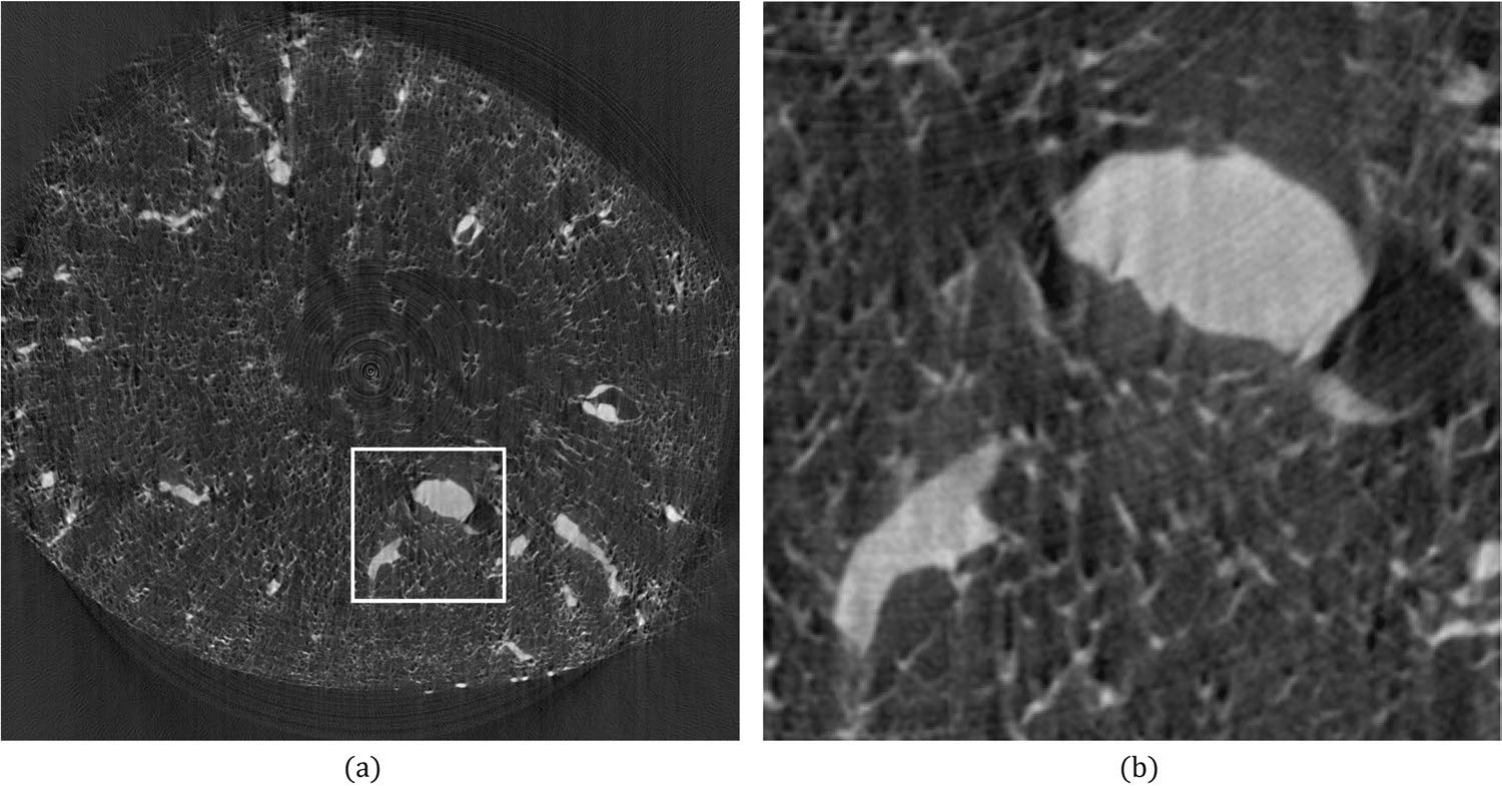
Tomographic images of mouse kidney reconstructed from 601 projections with 1600 parallel beams. The projection angle was configured to be 0.2995° for projection acquisition. (**a**) Reconstruction result without any correction. (**b**) Partial enlargements of the region bounded by the white-bordered rectangle in (**a**).

**Figure 5:**
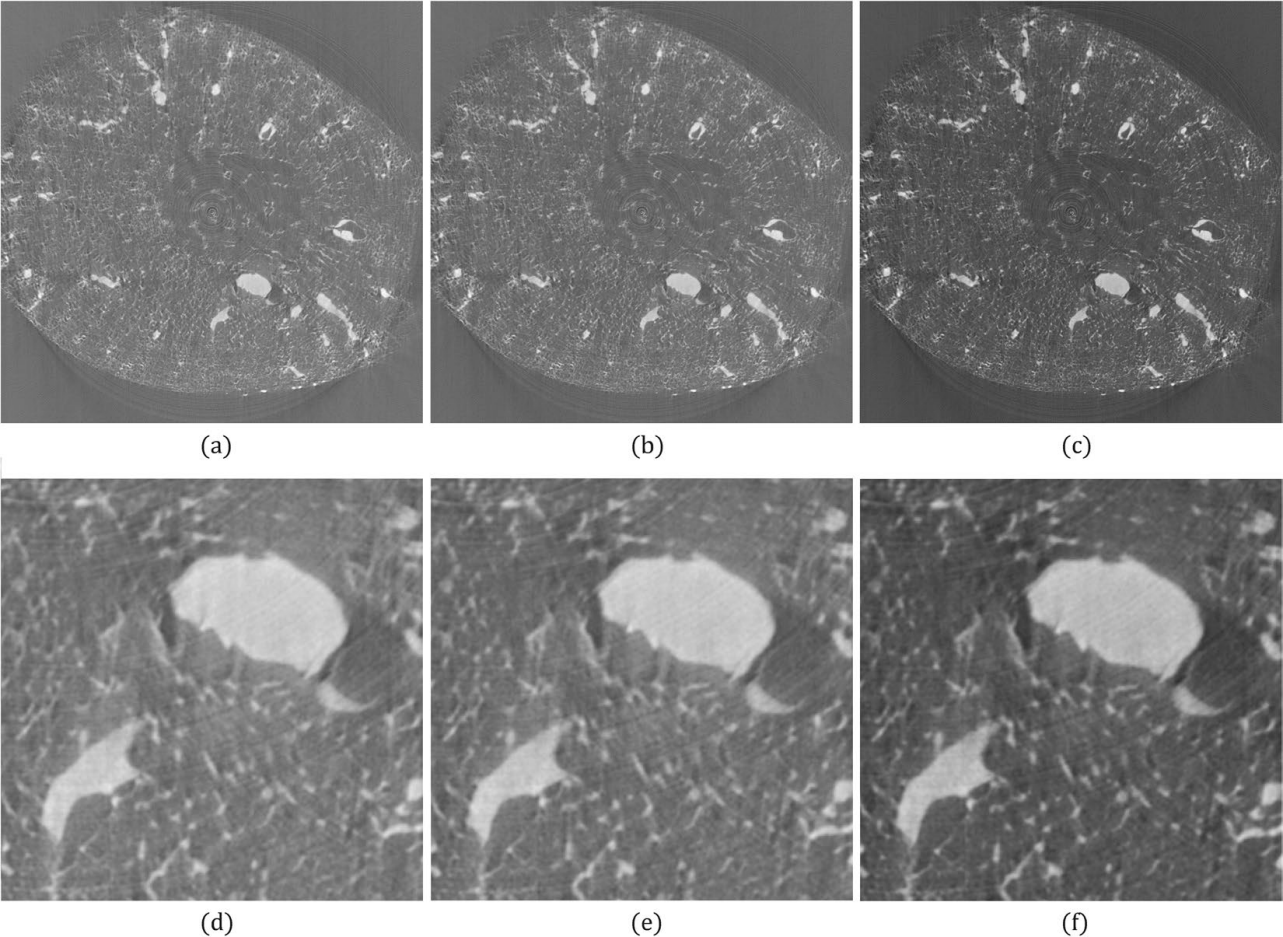
Reconstructed images of the same data in Fig. 4 after correcting errors. (**a**) Reconstruction result when *δ*_*B*_ was corrected by the ENP method. (**b**) Reconstruction result when *θ*_*E*_ was corrected by the FC method. (**c**) Reconstruction result when *δ*_*B*_ and *θ*_*E*_ were corrected by the proposed method. (**d**–**f**) Are the partial enlargements of the region in (**a**–**c**) respectively.

The proposed multi-range testing method was applied with *H* = 3, *α*_0_ = 1.0, and *t*_*m*_ = 20. *δ*_*B*_ was shifted toward the right by 3.75 pixels, and *θ*_*E*_ was corrected to 0.3005° (0.167%). The time required was 1807 s (approximately 18 s for a test). Figure 5c shows the image obtained using the proposed method. Figure 5f shows the enlarged view of Fig. 5c with the same view boundary as in Fig. 4a.

Because no ground truth was available for this data set, the MSE was not determined. Using a visual comparison of the three results could not indicate that the proposed method had the best result. This could be because the error with the projection angle was small (0.167%). However, the image reconstructed using the proposed method had the best contrast. From the experiments of the phantom data sets, we believe that our reconstructed image is closer to the true mouse kidney.

## Conclusion and Discussions

This paper presents a method for correcting both the rotational axis biases and projection angle errors. The proposed method uses *TV* as a metric for evaluating the quality of tomographic reconstruction. The gradient descent method is then applied to correct the errors.

The proposed method requires an adequate range of [**u**_*a*_, **u**_*b*_] and an initial point **u**^0^. Determining the most appropriate range and **u**^0^ for each case is difficult. We thus propose the method of multi-range testing to address this problem. In our experiments, a large range did not prevent the method from finding a solution nor increased the computing time substantially. Moreover, the differences between the *TV* values or MSE values (available for the phantom data experiments) were not significant for different ranges during the multi-range test. In particular, in experiments conducted using real images (mouse kidney experiments), the range was not sensitive to the reconstructed result. We conjectured that real images contain more information, and the gradient descent method is more robust if images contain more information.

We used Phantom 2 to validate this conjecture. Information complexity is defined as the entropy of an image in information theory^9^. If the entropy is normalized to the range [0, 1], an image is informative if its entropy is close to 1. A variation of Phantom 2 was created by blending two copies of Phantom 2 (Fig. 6a). Figure 6b shows the reconstructed image without any correction, and Fig. 6c presents the corrected image. The multi-range testing method was applied to both Phantom 2 and the variation of Phantom 2 by using the same set of parameters. The *TV* values obtained from all the ranges were normalized to the range [0, 1]. The histograms of the *TV* for both cases were constructed with a bin size of 0.2. Figure 7a,b illustrate the histograms of the *TV* values of the reconstructed Phantom 2 and its variation respectively. The variation of Phantom 2 contained more information, and more ranges fell within bin 1.

**Figure 6:**
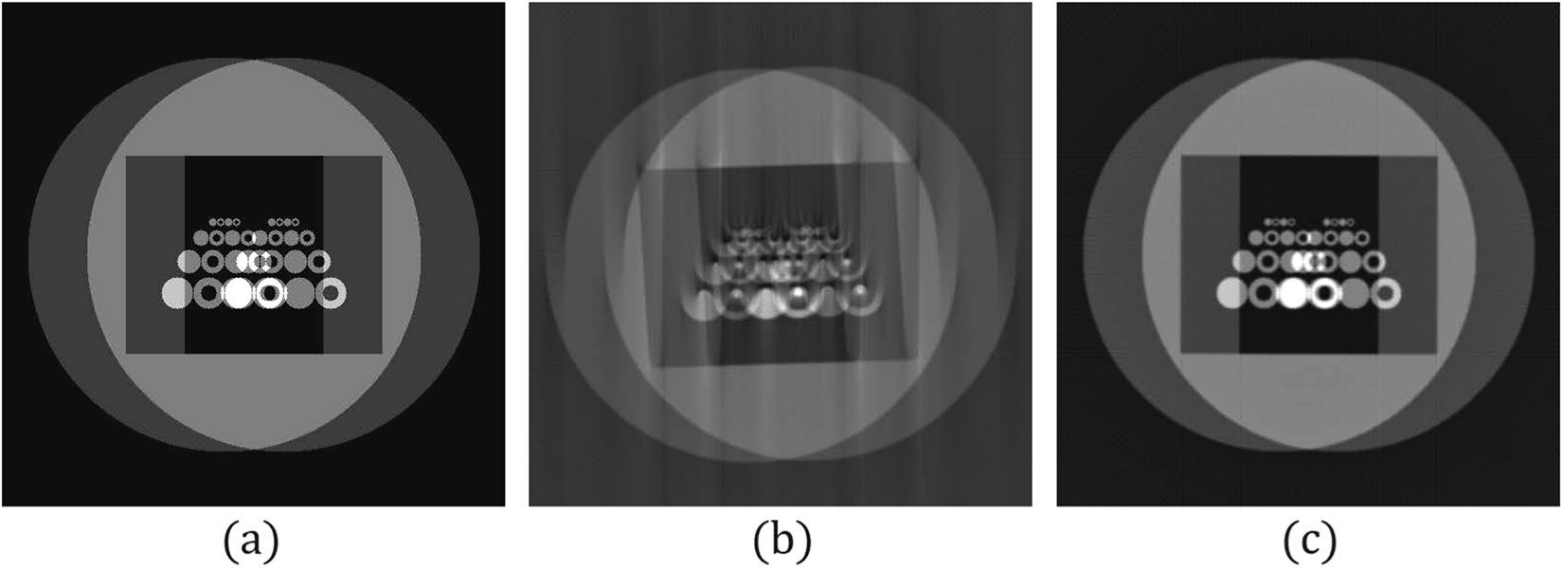
Experiment of the variation of the image of Phantom 2. This image contains more information than the Phantom 2 image. Tomographic images were reconstructed from 180 projections with 512 parallel beams. The interval of angles for generating the projections was 1.02° and the projected rotational axis was shifted to the left by 10 pixels. (**a**) Target image. (**b**) Reconstruction result without error corrections. (**c**) Reconstruction result obtained by the proposed method.

**Figure 7:**
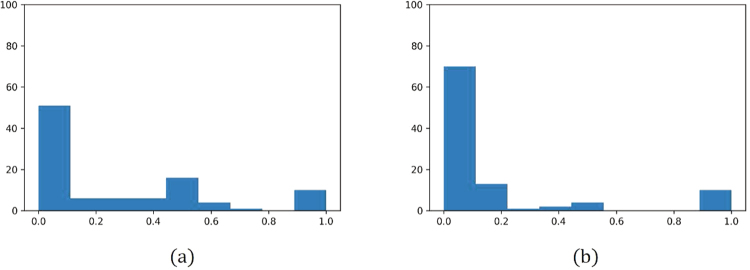
Comparison of the histograms of the total variations of the reconstructed images of Phantom 2 and the variation of the images Phantom 2. The total variations were normalized to [0.0, 1.0] and the histograms were constructed with a bin size of 0.2. (**a**) Histogram of Phantom 2. (**b**) Histogram of the variation of phantom 2.

For the case of the true image, the entropy of the image should be higher than 0.7, which is the entropy of the variation of Phantom 2. For example, the entropy of the reconstructed mouse kidney was 0.79; thus, more ranges could fall in the first bin. This conjecture suggests that multi-range testing may not be necessary. Randomly choosing, for example, three ranges from the range ±50 pixels for *δ*_*B*_ and ±10% for *θ*_*E*_ can result in a solution close to the best solution obtained by the multi-range testing.

The proposed method can efficiently compute *δ*_*B*_ and *θ*_*E*_ simultaneously and improve the quality of reconstructed images. From the study of the data sets Phantom 1 and Phantom 2, we believe that the proposed method can correct the errors for the reconstruction of real objects. We implemented the proposed method as a software system named *nct* that can be downloaded from the following link: http://www.cs.nctu.edu.tw/~chengchc/nct. The data sets used in this work can also be downloaded from the link.

## Electronic supplementary material

Implementation and test data
