# Supplementary material for: Correction of center of rotation and projection angle in synchrotron X-ray computed tomography
Source: Sci Rep. 2018 Jun 29;8:9884. doi: 10.1038/s41598-018-28149-8 (PMC6026166; doi:10.1038/s41598-018-28149-8)
Supplement: Supplementary file 1 — Implementation and test data [file 41598_2018_28149_MOESM1_ESM.pdf]

Correction of center of rotation and projection angle in synchrotron X-ray computed tomography

Chang-Chieh Cheng, Yu-Tai Ching, Pai-Hung Ko, and Yeukuang Hwu

Supplementary Information

1. Implementation

nct, NanoX Computed Tomography, v1.005

Windows 64-bit: http://people.cs.nctu.edu.tw/~chengchc/nct/nct_win64.zip

Mac OSX: http://people.cs.nctu.edu.tw/~chengchc/nct/nct_mac.zip

2. Test data

http://people.cs.nctu.edu.tw/~chengchc/nct/testdata.zip

Including Phantom1, Phantom2, and Mouse Kidney.
